# Knowledge about Cervical Cancer and Associated Factors among 15-49 Year Old Women in Dessie Town, Northeast Ethiopia

**DOI:** 10.1371/journal.pone.0163136

**Published:** 2016-09-30

**Authors:** Israel Mitiku, Fasika Tefera

**Affiliations:** 1 Department of Public Health, College of Medicine and Health Science, Wollo University, Dessie, Ethiopia; 2 Ethiopian Family Guidance Association, Addis Ababa, Ethiopia; National and Kapodistrian University of Athens, GREECE

## Abstract

**Background:**

Cervical cancer is one of the leading causes of morbidity and mortality amongst female cancer worldwide, especially in developing countries, including Ethiopia. The level of women’s knowledge about cervical cancer is not well documented in Ethiopia. The current study sought to assess women’s knowledge about cervical cancer and associated factors.

**Methods:**

A community based cross-sectional survey was conducted with a sample of 620 women aged 15–49 years residing in Dessie town, Northeast Ethiopia. Respondents were selected using a multistage sampling technique. The women were interviewed at home by trained data collectors using a structured questionnaire on cervical cancer knowledge. Knowledge about cervical cancer was measured using an eight item instrument. The maximum possible score was 8; those scoring 5 or more were categorized as having “sufficient” knowledge. Binary and multiple logistic regressions were employed to determine factors associated with knowledge about cervical cancer.

**Results:**

A total of 51% of the participants had sufficient knowledge about cervical cancer. After adjusting for covariates, having sufficient knowledge about cervical cancer was positively associated with better educational level and income. Women with primary education (Adjusted Odds Ratio (AOR): 3.4; 95% CI: 2.2–5.1) and those who had secondary and above education (AOR: 8.7; 95% CI: 5.5–13.7) were more likely to have sufficient knowledge about cervical cancer compared to those who had no formal education. Furthermore, women earning an average household monthly income above 1500 Ethiopian birr (ETB) (~75 U.S. dollars) were more likely to have sufficient knowledge (AOR: 2.3; 95% CI: 1.3–3.9) than women with an average household monthly income less than 500 ETB (~25 U.S. dollars).

**Conclusion:**

This study shows a suboptimal knowledge about cervical cancer regarding its risk factors, signs and symptoms, prevention and treatment among women in the study site. The level of education and economic status were found to be important determinants for knowledge about cervical cancer. Prevention programs should focus cervical cancer educational resources on women with less education and women with lower economic status groups.

## Background

Cervical cancer is a preventable disease, yet in 2012 it accounted for an estimated 527,624 newly diagnosed cases and 265,653 deaths worldwide [1]. The highest incidence of the disease is estimated to occur in developing countries including those in Sub-Saharan Africa (SSA) [1–3]. Ethiopia is one of the countries in SSA with the highest incidence and mortality rates for cervical cancer. In 2014, about 27.19 million women above the age of 15 years were estimated to be at risk of developing cervical cancer. Current estimates indicate that estimated 7,095 cases of cervical cancer and 4,732 deaths resulting from it occur every year [4].

Among all cancers, cervical cancer is the one which can be most effectively controlled by organised screening programmes [5]. Screening is a universally accepted early detection strategy that allows for treatment of precancerous lesions before they progress to invasive cancer [6–8]. Declines in cervical cancer incidence and mortality observed in high income countries have been reported to be related to the implementation of national screening programs [9, 10]. Although cervical cancer can be easily detected at an early precancerous stage, most women in SSA only seek treatment and care in an advanced stage of the disease [11, 12]. The offering and utilization of screening in many developing countries including Ethiopia is still poor [13, 14]. Currently, there is only one cervical cancer treatment in Ethiopia. The government of Ethiopia working to establish additional comprehensive cancer centers and expand see and treat cervical cancer programs. Several factors contribute to inefficient screening of cervical cancer and determine the stage at patients with cervical cancer present to the health facility in low income countries [15]. Nonexistence of a national screening system, and the low access of the impoverished population to health care have been reported to contribute to inefficient testing, late diagnosis and late treatment [16, 17]. Moreover, previous studies have reported that women’s knowledge about cervical cancer can affect cervical cancer screening participation [16, 18].

Though cervical cancer is a major public health problem facing women in Ethiopia, there are limited data on women’s knowledge of cervical cancer and associated factors. Thus, the objectives of this study were to determine women’s knowledge about cervical and identify socio-demographic factors related to knowledge about cervical cancer. The findings of this study inform policy to design targeted and tailored strategies to increase cervical cancer knowledge and potentially increase cervical cancer screening uptake among Ethiopian women.

## Methods

### Study design

A community based cross-sectional study was conducted among women of reproductive age (15–49 years) in Dessie town in January 2015. Dessie town is located 401 km Northeast of Addis Ababa (the capital city of Ethiopia). The total population of the town in 2015 as projected based on the 2007 census was 187,917 [19].

### Sample size and sampling procedure

The sample size calculation was based on a single population proportion formula. Because of the unavailability of recent information about the topic, sample size was calculated taking prevalence of sufficient knowledge to be 50%. Assuming a 95% confidence level and 5% margin of error, the minimum sample size required for the study was 634 after including a design effect of 1.5 and 10% non-response rate.

A multistage sampling technique was used to select women. First, five kebeles were selected using simple random sampling from ten kebeles in the town. A kebele is the smallest administrative unit in Ethiopia. A sampling frame for list of households with women of reproductive age from each selected kebele were prepared in consultation with health extension workers and local administrators. Then the total sample size was allocated proportional to the size of the women of reproductive age at each Kebele. Finally, systematic random sampling method was used to select households and women for an interview. The women were included if they were aged 15–49 years.

### Data collection

A pre-tested structured questionnaire initially developed in English and then translated into the local language (Amharic) was used for this study. Then backward translation from Amharic to English was performed to verify the accuracy of the translation. The questionnaire was designed based on the study objectives, taking help from the previous literature and studies available on the topic added with content specific questions.

The questionnaire included socio-demographic characteristics and questions regarding the knowledge about different aspects of cervical cancer. Trained health extension workers administered a questionnaire using face-to-face interviews. The data collection was supervised by trained supervisors. All completed questionnaires were checked by supervisors for completeness and consistency at field level.

### Measures

Knowledge about cervical cancer was measured using an eight item instrument adapted from a previous study in the Democratic Republic of Congo [20]. For each item, a participant’s answer was considered to be correct if she gave one of the expected answers. Scores were computed by taking the sum of correct responses, resulting in scores ranging from 0 to 8. To facilitate comparison, the women’s score on knowledge was converted to a binary variable. Women with a score of 5 or more were categorized as having “sufficient” knowledge; the others were categorized as having “no sufficient” knowledge. The reliability of items of the scale was evaluated using Cronbach’s alpha. A cut-off value of 0.7 and above was used as acceptable internal consistency level [21], and we found a high internal consistency in the present study (Cronbach’s α = 0.95)

### Data analysis

Data were entered and cleaned using EPi-Info version 3.5.2 and was exported to SPSS version 20 for analysis. Descriptive statistics were used to summarize all variables of interest in the study population. The statistical association between the outcome variable (having sufficient knowledge about cervical cancer) and the explanatory variables were first tested using binary logistic regression. Multivariable logistic regression was used to control confounding effects. Both backwards and forward logistic regression was performed and gave similar result. The results of all logistic regression analyses are reported as odds ratios (OR) with 95% confidence intervals (CIs). The final multivariate model was tested for goodness of fit with the Hosmer-Lemeshow test. P-values ≤0.05 were considered statistical significant.

### Ethical Considerations

The study was approved by the Ethics Committee of the College of Medicine and Health Sciences of Wollo University. The purpose and importance of the study was explained to participants and verbal informed consent was obtained from every study participant using consent card. Consent was received from adult next-of-kin when the respondent was under 18 years of age. Confidentiality of the information was maintained throughout the study by excluding personal identifiers from the data collection form.

## Results

### Socio demographic characteristics of the study population

A total of 620 women were interviewed; the response rate was 97.8%. Five hundred twenty-three women (84.3%) were aged between 25–49 years. Most of the respondents (61.6%) were married, and 76.0% had one or more children. Of the participants, (58.2%) completed secondary school and (46.9%) have no formal employment and were housewives. Around half (50.8%) of the participants were Orthodox Christians while 249 (45.0%) were Muslims (Table 1).

**Table 1:** Socio-demographic characteristics of respondents, Dessie town, Ethiopia, 2015.

**Characteristic**	**Frequency**	**Percent**
**Age**		
15–24	97	15.7
25–34	324	52.3
35–49	199	32.0
**Religion**		
Orthodox Christian	315	50.8
Muslim	279	45.0
Protestant Christian	18	2.9
Catholic Christian	8	1.3
**Educational level**		
No formal education	262	42.3
Primary	156	25.2
Secondary and above	202	32.5
**Occupation**		
Employed	231	37.7
Housewife	291	46.9
Merchant	47	7.6
Student	51	8.2
**Marital status**		
Married	382	61.6
Single	127	20.5
Divorced/Separated	77	12.4
Widowed	34	5.5
**Parity**		
0	149	24.0
1–4	432	69.7
≥5	39	6.3
**Average monthly household income (ETB)**		
< 500 (~25 USD)	133	21.5
500–1000 (~25–50 USD)	197	31.8
1000–1500 (~50–75 USD)	130	21.0
>1500 (~75 USD)	160	25.8

ETB: Ethiopian birr

USD: U. S. dollars

### Awareness of women about cervical cancer

The women were first asked if they have ever heard of cervical cancer. Three hundred fifty-eight (57.7%) of the women reported that they had heard about cervical cancer. Media (radio/television) was the predominant (55.3%) source of information followed by health care providers (33.0%).

### Knowledge about the risk factors and presenting symptoms of cervical cancer

To assess their knowledge about the risk factors of cervical cancer, participants were asked, whether human papilloma virus (HPV), multiple sexual partners, early onset of sexual activity, and/or cigarette smoking could cause or promote cervical cancer. About 58.1% of the respondents did not know whether there are risk factors for cervical cancer or not. Two hundred and sixty (41.9%) of the participants were able to identify at least one risk factor for cervical cancer (Table 2). Multiple sexual partners and sexually transmitted infections (STI) were specific risk factors mentioned by 134 (21.6%) and 67 (10.8%) of the respondents respectively.

**Table 2:** Knowledge of women about risk factors, presenting symptoms, prevention measures and treatment options of cervical cancer, Dessie town, Ethiopia, January 2015.

**Questions**	**Expected response(s)**	**Number**	**Percent**
Ever heard of cervical cancer	Yes	358	57.7
Symptoms			
	Vaginal bleeding, vaginal discharge	287	46.3
Risk factors of cervical cancer	Multiple sexual partner, early sex, HPV, cigarette smoking	260	41.9
Cervical cancer can be cured if detected in its early stage	Yes	334	53.9
Treatment options	Surgery, chemotherapy, radiotherapy	326	52.6
Preventive options	Avoid early sex, avoid multiple sexual partners, quit smoking, vaccination	358	57.7
Treatment options	Surgery, chemotherapy, radiotherapy	326	52.6
Ever heard about cervical screening	Yes	341	55

Regarding signs and symptoms of cervical cancer, 46.3% of the respondents mentioned either vaginal bleeding or foul smelling vaginal discharge as symptoms of the disease. However, 333 (53.7%) of the respondents did not know any symptom.

### Knowledge of women about prevention measures and treatment options of cervical cancer

Two hundred fifty-five (41.1%) of the respondents knew that cervical cancer can be prevented. Reduced number of sexual partners was mentioned by 150 (24.2%) of the respondents as a helpful prevention measure. Other stated prevention methods were avoiding early coitus and cigarette smoking. However, 365 (58.9%) of the respondents did not know any cervical cancer prevention measure. More than half (55%) of participants had ever heard of cervical screening. 52.6% of the respondents knew that cervical cancer can be treated using chemotherapy, surgery or radiotherapy.

### Overall knowledge about cervical cancer and associated factors

Using the sum of all knowledge items, we determined that a total of 51% of the participants had sufficient knowledge about cervical cancer.

On bivariate analysis, six socio-demographic characteristics were found to be significantly associated with having sufficient knowledge about cervical cancer: age, educational level, marital status, occupation, average monthly income and parity. Further analysis to determine association between knowledge about cervical cancer and variables that were found to have positive association in binary test shows that only educational level and average monthly income were found to have significant association with level of knowledge about cervical cancer. Women who had some form of education were more likely to have sufficient knowledge about cervical cancer. Women with primary education (AOR: 3.4; 95% CI: 2.2–5.1) and those who had secondary and above education (AOR: 8.7; 95% CI: 5.5–13.7) were more likely to have sufficient knowledge about cervical cancer. Similarly, women who reported an average household monthly income above 1500 Ethiopian birr (ETB) (~ 75 U. S. dollars (USD)) were 2.3 times more likely to have sufficient knowledge than women with an average household monthly income less than 500 ETB (~25 USD) (AOR: 2.3; 95% CI: 1.3–3.9) (Table 3).

**Table 3:** Factors associated with knowledge about cervical cancer among women aged 15–49 years in Dessie town, Ethiopia, January 2015.

**Variables**	**Sufficient knowledge about cervical cancer**		**COR (95% CI)**	**AOR (95% CI)**
	Yes *(n*, %)	No (*n*, %)		
**Age**				
15–24	50 (51.5)	47 (48.5)	1.3 (0.8–2.2)	
25–34	178 (54.9)	146 (45.1)	**1.5 (1.1–2.2)**	
35–49	88 (44.2)	111 (55.8)	1.00	
**Educational level**				
No formal education	69 (26.3)	193 (73.7)	1.00	1.00
Primary	86 (55.1)	70 (44.9)	**3.4 (2.3–5.2)**	**3.4 (2.2–5.1)**
Secondary and above	161 (79.7)	41 (20.3)	**11.0 (7.1–17.0)**	**8.7 (5.5–13.7)**
**Occupation**				
Housewife	123 (42.3)	168 (57.7)	1.00	
Merchant	27 (57.4)	20 (42.6)	1.8 (1.0–3.4)	
Student	29 (56.9)	22 (43.1)	1.8 (1.0–3.3)	
Employed	137 (59.3)	94 (40.7)	**2.0 (1.4–2.8)**	
**Parity**				
0	92 (61.7)	57 (38.3)	1.00	
1–4 children	213 (49.3)	219 (50.7)	**4.1 (1.9–8.9)**	
≥5	11 (28.2)	28 (71.8)	**2.5 (1.2–5.1)**	
**Marital status**				
Married	197 (52.6)	185 (47.4)	1.4 (0.9–2.1)	
Single	76 (59.8)	51 (40.2)	1.00	
Divorced/Separated	30 (39.0)	47 (61.0)	**0.4 (0.2–0.7)**	
Widowed	13 (38.2)	21 (61.8)	0.6 (0.3–1.2)	
**Average monthly household income (ETB)**				
<500	64 (48.1)	69 (51.9)	1.00	1.00
500–1000	66 (33.5)	131 (66.5)	0.5 (0.3–0.9)	0.9 (0.5–1.5)
1001–1500	71 (54.6)	59 (45.4)	1.2 (0.8–2.1)	1.4 (0.8–2.3)
>1500	115 (71.9)	45 (28.1)	2.8 (1.7–4.5)	**2.3 (1.3–3.9)**

Multivariable model adjusted for all variables. COR: crude odds ratio; AOR: adjusted odds ratio. Values in bold are statistically significant at P ≤0.05.

## Discussion

Cervical cancer screening is a critical and the most effective method for early detection and treatment of precancerous lesions and mortality reduction of cervical cancer. The present study was conducted to determine the level of knowledge about cervical cancer among women in Dessie town, Ethiopia. The study also intended to identify socio-demographic factors influencing women’s knowledge about cervical cancer. Our results demonstrate that only 51.9% of women had sufficient knowledge on risk factors, signs and symptoms, preventive and treatment methods of cervical cancer. Women’s educational level and average monthly household income were found to affect their knowledge about cervical cancer.

In this study, 57.7% of women answered to have heard about cervical cancer. These results are lower than those published for Ethiopia (78.7%) [22], Democratic Republic of Congo (82%) [20], and Nigeria (71%) [23]. Regarding the risk factors, about 42% of the study participants were able to identify one or more correct risk factors for cervical cancer like STIs, early onset of sexual activity, multiple sexual partners and smoking, which is considerably higher than the Fig described by a study carried out in Gondar, Ethiopia (31.0%) [22] and Democratic Republic of Congo (19.3%) [20]. Higher Figs have been published for South Africa where 64.0% of the respondents gave at least one correct risk factor for cervical cancer [14]. The discrepancy might be attributed to the fact that South Africa has a national cervical cancer screening policy.

Overall, 46.1% of the respondents mentioned either vaginal bleeding or foul smelling vaginal discharge as symptoms of cervical cancer. However, 43.9% of the respondents did not know any symptom. This finding is consistent with a publication from Gondar, Ethiopia, which showed that 39.6% did not know any symptoms. Two hundred fifty-five (41.1%) of the respondents knew that cervical cancer can be prevented, which is higher that the Fig reported from the Democratic Republic of Congo (17.6%) [20]. Higher Figs have been published for Gondar, Ethiopia where 63.9% of the respondents were found to be aware that cervical cancer can be prevented. Concerning treatment, 52.6% of the respondents knew that cervical cancer can be treated, which is slightly lower compared to another study from Ethiopia (66.1%) [22]. The proportion of women who had heard about cervical screening found in this study (55%) is substantially higher than the Fig described by a study carried elsewhere in Africa [20].

Overall, the current study showed that only 51% of respondents had sufficient knowledge on risk factors, signs and symptoms, preventive and treatment methods for cervical cancer. Lower Figs have been published for Tanzania (19.2%) [16] and in other similar report for Ethiopia (31.1%) [22]. Differences in the measurement of “knowledge” about cancer make the comparison difficult. The findings indicate that the level of knowledge about cervical cancer is suboptimal and of concern as women’s knowledge plays a role in rate of screening uptake [16]. Having full information about cervical cancer and its prevention has been reported to give women the ability to make an informed decision to undergo screening [24].

This study also found that level of education was significantly associated with women’s knowledge about cervical cancer. Women with primary, and secondary and above level of education were more likely to have sufficient knowledge about cervical cancer screening than those who had no formal education; a phenomenon observed in other studies [22, 20].

Results further showed that average monthly household income differed significantly between those women who had sufficient knowledge about cervical cancer versus who had no sufficient knowledge. Women with an average monthly household income above 1500 ETB (~75 USD) were more likely to have sufficient knowledge than women with less than 500 ETB (~25 USD) income. It is possible that increased socio-economic status place the women in a better position economically and knowledge wise. Studies have also reported monthly income to significantly determine uptake of cervical screening [25, 26]

## Limitations

The study was conducted in an urban setting, which may limit generalizability of the findings to all women in Ethiopia, particularly to those in rural areas.

## Conclusions

Our findings underline that the level of knowledge about cervical cancer regarding its risk factors, signs and symptoms, prevention and treatment is suboptimal among the population under study. The level of education and economic status were found to be important determinants for knowledge about cervical cancer. Prevention programs should focus on cervical cancer educational resources on women with less education and women in lower economic status groups.

## Supporting Information

S1 Table Questonaire. (DOCX)
