# Supplementary material for: Knowledge about Cervical Cancer and Associated Factors among 15-49 Year Old Women in Dessie Town, Northeast Ethiopia
Source: PLoS One. 2016 Sep 30;11(9):e0163136. doi: 10.1371/journal.pone.0163136 (PMC5045174; doi:10.1371/journal.pone.0163136)
Supplement: S1 Table — (DOCX) [file pone.0163136.s001.docx]

## Questionnaire (English version)

**Part 1. Socio-demographic characteristics**

| **S. No** | **Questions** | **Coding classifications** |
| --- | --- | --- |
| 101 | How old are you? | _____________ years |
| 102 | What is your occupation at this time? | 1. Government employed 2. Housewife 3. Merchant 4. Student 5. Other, specify_________ |
| 103 | What is the highest level of education you reached? | 1. Cannot read and write 2. Read and write only 3. Primary school 4. Secondary school and above |
| 104 | What is your religion? | 1. Orthodox 2. Muslim 3. Protestant 4. Catholic 5. Others |
| 105 | What is your marital status? | 1. Single 2. Married 3. Divorced 4. Widowed |
| 106 | Parity | ___________ |
| 107 | What is your average monthly household income? | ____________ ETB |

**Part 2: Knowledge on cervical cancer**

| 201 | Have you ever heard about cervical cancer | 1. Yes 2. No |
| --- | --- | --- |
| 202 | Where did you first learn about carcinoma of the cervix? (More than one answer is allowed) | 1. News Media 2. Brochures, posters and other printed materials 3. Health workers 4. Family, friends, neighbors and colleagues 5. Religious leaders 6. Teachers 7. Other, specify ________________ |
| 203 | What are the symptoms of carcinoma of the cervix? (More than one answer is allowed) | 1. Vaginal bleeding 2. Vaginal foul smelling discharge 3. Do not know 4. Other, specify _______________ |
| 204 | What are the risk factors for cancer of the cervix? (More than one answer is allowed) | 1. Having multiple sexual partners 2. Early sexual intercourse 3. Human papilloma virus 4. Cigarette smoking 5. Do not know 6. Other, specify ______________ |
| 205 | How can a person prevent getting cancer of the cervix? (More than one answer is allowed) | 1. Avoid multiple sexual partners 2. Avoid early sexual intercourse 3. Quit cigarette smoking 4. HPV vaccination 5. Do not know 6. Other, specify ______________ |
| 206 | Can cancer of the cervix be cured in its earliest stages? | 1. Yes 2. No 3. Don’t know |
| 207 | How can someone with cancer of the cervix be treated? (More than one answer is allowed) | 1. Surgery 2. Specific drugs given by hospital 3. Radiotherapy 4. Do not know 5. Other, specify ___________ |
| 208 | Have you ever heard about cervical cancer screening? | 1. Yes 2. No |

**This is the end of the questionnaire. Thank you very much for taking time to answer these questions.**

## Amharic questionnaire

| **ተ.ቁ** | | **ጥያቄወች** | **መሌስ ሉሆኑ የሚችለ ዝርዝሮች** | **ይለፉ** | **የመልስኮድ** |  |
| --- | --- | --- | --- | --- | --- | --- |
| **ክፍል 1፡ የማህበራዊ ና የስነ-ህዝብ ገጽታወች** | | | | | |  |
| 101 | | ዕዴሜዎ ስንት ነው? | ___________ ዓመት |  | | |
| 102 | | ስራዎት ምንድን ነው? | 1. የመንግስት ሰራተኛ 2. የቤትእመቤት 3. ነጋዴ 4. ተማሪ 5. ሌላ ካለ ይጠቀስ ____________ |  | | |
| 103 | | የትምህርት ደረጃዎ ምን ያህሌ ነው? | 1. ማንበብና መጻፍ የማይችል 2. ማንበብና መጻፍ ብቻ የሚችል 3. የመጀመሪያ ደረጃ 4. ሁለተኛ ደረጃ ና ከዛበላየ |  | | |
| 104 | | ሐይማኖተዎ ምንዴን ነው? | 1. ኦርቶደክስ 2. ሙሰሊም 3. ፕሮቴስታንት 4. ካቶሊክ 5. ሌላ ካለ ይጠቀሰ __________ |  | | |
| 105 | | የጋብቻሁኔታ? | 1. ያገባች 2. ያላገባች 3. የተፋታች 4. ባል የሞተባት |  | | |
| 106 | | ስንት ልጆች ወልደዋል? | ____________ |  | | |
| 107 | | በአማካይ የቤተሰብዎ የወር ገቢ ምን ያህል ነው ? | _____________ ብር |  | | |
| **ክፍል 2. ስለማህጸን በር ካንሰር የእውቀት ዳሰሳ** | | | | | |  |
| 201 | ስለማህጸን በር ካንሰር ሰምተሽ ታውቂያለሽ? | | 1. አዎ 2. አላውቅም |  | | |
| 202 | ስለማህጸን በር ካንሰር ለመጀመሪያ ጊዜ ከየት ሰማሸ | | 1. ከዜና ማሰራጫ 2. ከበራሪ ወረቀት እና ከማስተማሪያ ጽሁፎች 3. ከጤና ባለሞያ 4. ከቤተሰብ, ከጓደኛ 5. ከሀይማኖት አባቶች 6. ከመምህራን 7. ሌላ ካለ ይገለጽ __________ |  | | |
| 203 | የማህጸን በር ካንሰር ምልክቶች ምንድንናቸው | | 1. በብልት ደም መፍሰስ 2. በብልት ሸታ ያለው ፈሳሽ መፍሰስ 3. አላውቅም 4. ሌላ ካለ ይገለጽ __________ |  | | |
| 204 | ለማህጸን በር ካንሰር አጋላጭ ሁኔታዎች ምንድንናቸው | | 1. ከአንድ በላይ የወሲብ ጎደኛ መኖር 2. ቀድሞ የወሲብ ግንኘነት መጀመር 3. በበሸታ አምጭ ተዋስያን 4. ሲጋራ በማጨስ 5. አላውቅም 6. ሌላ ካለ ይገለጽ __________ |  | | |
| 205 | የማህጸን በር ካንሰርን እንዴት መከላከል ይቻላል ብለው ያስባሉ | | 1. የወሲብ ጓዳኝን በመቀነስ 2. ቀድሞ የወስብ ግንኘነትን ባለመጀመር 3. ባለማጨስ 4. ክትባት በመውሰድ 5. አላውቅም 6. ሌላ ካለ ይገለጽ __________ |  | | |
| 206 | የመጀመሪያ ደረጃ የማህጸን በር ካንሰር ይድናል ብለው ያስባሉ | | 1. አዎ 2. የለም 3. አላውቅም |  | | |
| 207 | በሽታው ያለበት ሰው በምን ሊታከም ይችላል ብለው ያስባሉ | | 1. በቀዶ ጥገና 2. የህክምና መድሃኒት በመውሰድ 3. በጨረርህክምና 4. አላውቅም |  | | |
| 208 | ስለማህጸን በር ካንሰር ቅድመ ምርመራ ሰምተሽ ታውቂያለሸ | | 1. አዎ 2. አላውቅም |  | | |

ቃለመጠይቁን ጨርሰናሌ፡፡ ላደረጉልን ትብብር ከልብ እናመሰግናለን፡ ፡
